# Long-Term *in vivo* Release Profile of Dexamethasone-Loaded Silicone Rods Implanted Into the Cochlea of Guinea Pigs

**DOI:** 10.3389/fneur.2019.01377

**Published:** 2020-01-22

**Authors:** Arne Liebau, Sören Schilp, Kenneth Mugridge, Ilona Schön, Michel Kather, Bernd Kammerer, Jochen Tillein, Susanne Braun, Stefan K. Plontke

**Affiliations:** ^1^Department of Otorhinolaryngology, Head and Neck Surgery, Martin Luther University Halle-Wittenberg, Halle, Germany; ^2^MED-EL Headquarters, Innsbruck, Austria; ^3^Center for Biological Systems Analysis ZBSA, Albert-Ludwigs-University Freiburg, Freiburg, Germany; ^4^Hermann Staudinger Graduate School, University of Freiburg, Freiburg, Germany; ^5^MED-EL Deutschland GmbH, Starnberg, Germany

**Keywords:** cochlear implant, insertion trauma, intracochlear drug delivery, dexamethasone, inner ear

## Abstract

Glucocorticoids are used intra-operatively in cochlear implant surgeries to reduce the inflammatory reaction caused by insertion trauma and the foreign body response against the electrode carrier after cochlear implantation. To prevent higher systemic concentrations of glucocorticoids that might cause undesirable systemic side effects, the drug should be applied locally. Since rapid clearance of glucocorticoids occurs in the inner ear fluid spaces, sustained application is supposedly more effective in suppressing foreign body and tissue reactions and in preserving neuronal structures. Embedding of the glucocorticoid dexamethasone into the cochlear implant electrode carrier and its continuous release may solve this problem. The aim of the present study was to examine how dexamethasone concentrations in the electrode carrier influence drug levels in the perilymph at different time points. Silicone rods were implanted through a cochleostomy into the basal turn of the scala tympani of guinea pigs. The silicone rods were loaded homogeneously with 0.1, 1, and 10% concentrations of dexamethasone. After implantation, dexamethasone concentrations in perilymph and cochlear tissue were measured at several time points over a period of up to 7 weeks. The kinetic was concentration-dependent and showed an initial burst release in the 10%- and the 1%-dexamethasone-loaded electrode carrier dummies. The 10%-loaded electrode carrier resulted in a more elevated and longer lasting burst release than the 1%-loaded carrier. Following this initial burst release phase, sustained dexamethasone levels of about 60 and 100 ng/ml were observed in the perilymph for the 1 and 10% loaded rods, respectively, during the remainder of the observation time. The 0.1% loaded carrier dummy achieved very low perilymph drug levels of about 0.5 ng/ml. The cochlear tissue drug concentration shows a similar dynamic to the perilymph drug concentration, but only reaches about 0.005–0.05% of the perilymph drug concentration. Dexamethasone can be released from silicone electrode carrier dummies in a controlled and sustained way over a period of several weeks, leading to constant drug concentrations in the scala tympani perilymph. No accumulation of dexamethasone was observed in the cochlear tissue. In consideration of experimental studies using similar drug depots and investigating physiological effects, an effective dose range between 50 and 100 ng/ml after burst release is suggested for the CI insertion trauma model.

## Introduction

Cochlear implants (CI) are successfully used to restore hearing in patients with profound sensory hearing loss in the entire frequency range or in the high frequencies from approximately 1 kHz (i.e., partial deafness). By introducing the strategy of combined electric and acoustic stimulation (EAS) in partial deafness hearing loss in the high and mid-frequency ranges is compensated by electrical stimulation whereas residual hearing in the low frequency range is supported by acoustic amplification (1, 2).

The function of the cochlear implant is based on stimulation of spiral ganglion cells which is why their preservation is decisive for the subsequent quality of hearing and rehabilitation success. However, even a low-trauma insertion of the electrode carrier into the cochlea might lead to mechanical trauma of the inner ear structures which induces inflammation and wound healing processes (3, 4). This may result in apoptosis of neuronal structures of the inner ear i.e., hair cells and spiral ganglia (5–7). Furthermore, for EAS hearing prostheses, the survival of cochlear structures and of hair cells responsible for low-frequency hearing is supported by the development of low-trauma electrode carriers and introduction of atraumatic insertion techniques (8, 9).

The inflammation and immune response caused by insertion trauma and a foreign body response against the electrode carrier induce the formation of fibrotic tissue and ossification within the scala tympani (3, 10). Encapsulation of the electrode carrier with fibrotic tissue results in an electrical isolation which increases the impedance of electrodes. Therefore, higher currents are needed to stimulate spiral ganglia which leads to increased energy consumption by the implant and a decreased dynamic range since the stimulation device must be driven closer to saturation (11). In addition, the higher voltages generated at the electrode contacts lead to spatial expansion of stimulation, and consequently to less frequency-specific stimulation.

There is increasing evidence from histopathological studies that delayed loss of low-frequency residual hearing under EAS conditions is not a consequence of inflammation-induced apoptosis of the hair cells or spiral ganglia. Rather, it is the result of fibrosis and ossification arising from of the foreign body response against the electrode carrier (12–16). This may explain the functional hearing loss which can occur months or even years after implantation. The mechanical properties of the basilar membrane and the microarchitecture of the organ of Corti might be compromised by the formation of fibrotic tissue and ossification of the cochlea (17). In addition, the normal pressure ratios within the inner ear ducts could be disturbed, especially when the small linking canals between the fluid-filled compartments in the inner ear like the cochlea aqueduct or the ductus reuniens are blocked by newly formed tissue (5, 16, 18).

The therapeutic indication for cochlear implantation is also currently expanding for patients for whom no atraumatic surgery is possible. For example, when an intralabyrinthine schwannoma tumor grows in the cochlea, it is removed by a partial or subtotal cochleoectomy while maintaining the modiolus or at least the modiolus of the basal and second turn in most cases. A perimodiolar CI electrode is then placed around the remaining modiolus for hearing rehabilitation afterwards. The preservation of the residual spiral ganglia in these patients is of particular importance for rehabilitative success (19).

Many experimental studies in animals have demonstrated that the use of corticosteroids is able to preserve hearing thresholds, increase the survival of hair cells and spiral ganglia, and decrease the formation of new fibrotic tissue within implanted cochlea (20–27). Clinical studies demonstrate lower impedances and improved preservation of low-frequency residual hearing in patients when corticosteroids are used in CI surgery (28–34). The efficacy is attributed to the anti-apoptotic and anti-inflammatory properties of glucocorticoids, by inhibiting immune cells and decreasing the release of inflammatory factors. In addition, stabilized homeostasis and ion balance, as well as increased blood flow, may contribute to the protective effects of glucocorticoids [for an overview see (5, 35, 36)].

Data from two published animal studies favor the local application of glucocorticoids over systemic application in order to minimize the consequences of insertion trauma (26, 27). On the contrary, results of another study showed better hair cell protection after local application but a more robust prevention of fibrosis for systemic administration (37). Results of clinical pilot studies considering pure tone audiometry or electrode impedance measurement showed a slight tendency in favor for local application but only included few patients (32, 33). Additionally, a prolonged application after insertion seems to be more effective in suppressing adverse effects than single-shot glucocorticoid application prior to insertion of the cochlear implant electrode (22–27). In a guinea pig model of insertion trauma, the measurements of inflammatory cytokines in perilymph revealed that suppression of inflammation is most successful when glucocorticoids are applied intracochlearly (26). However, due to the fast clearance of glucocorticoids in the inner ear fluid spaces achieving constant drug levels over a certain time period in local application is only feasible with sustained administration of the drug (38, 39). Taken together, these data strongly support the approach that uses the electrode carrier as a drug reservoir to realize long-term intracochlear corticosteroid application to the inner ear over the course of several weeks after implantation (40, 41).

An obvious strategy for using the electrode carrier as a drug delivery device is to incorporate drugs into the silicone of the carrier or to coat the electrode with a mixture of silicone and drug(s). This was addressed by several studies, but the resulted drug concentrations were unmeasured, or were measured for only a short time period (i.e., a few days after implantation) (42–47). For developing such drug-releasing electrode carriers, knowledge of pharmacokinetics is crucial to determine the correct drug load to achieve effective drug concentrations within the inner ear. In the present study, electrode carrier dummies with incorporated dexamethasone were tested in a guinea pig model of implantation. Three different dexamethasone concentrations were applied in 10-fold increasing steps, and the resulting drug concentration in perilymph was examined for up to 7 weeks after implantation.

## Materials and Methods

### Study Design

The aim of the study was to investigate the long-term pharmacokinetics of electrode carrier dummies (i.e., silicone rods loaded with dexamethasone), which were implanted into the scala tympani of guinea pigs. Three different dexamethasone concentrations were investigated to examine the time course of drug release into the perilymph and its dependency on drug load. Dexamethasone concentrations in the perilymph and cochlear tissue were measured at several time points for up to 7 weeks after implantation. All experiments were conducted in accordance with current regulations of the German Animal Welfare Law and were approved by the Saxony-Anhalt state office for consumer protection and veterinary affairs (reference 42502-2-1207MLU).

### Dexamethasone-Releasing Silicone Rods

**Figure 1:**
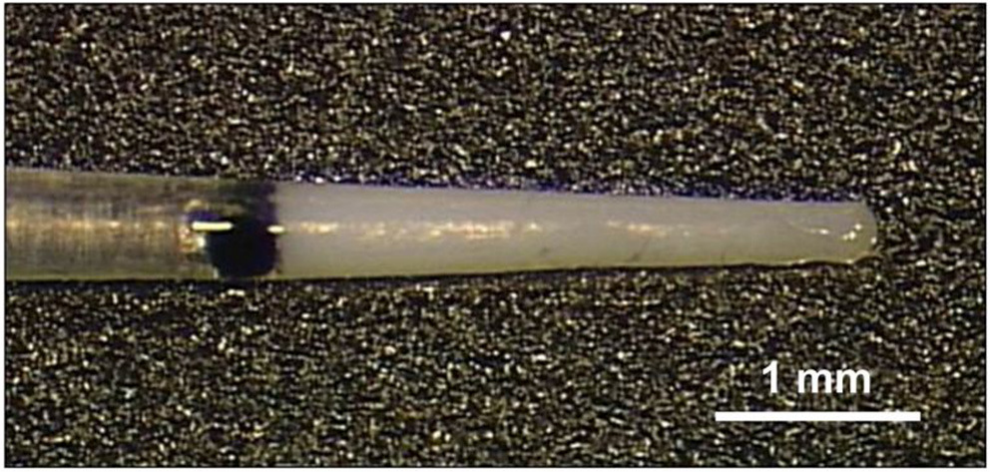
Silicone rod containing 10% (55 μg) dexamethasone. The drug is incorporated into the first 3 mm from the tip. The rod was implanted into the scala tympani. The black dot (colored silicone) indicates the insertion depth of 3 mm and was located at the rim of the cochleostomy.

Silicone rods were manufactured using medical grade liquid silicone rubber for cochlear implants (MED-4244, NuSil, USA). The dimensions of the custom-made rods were adapted to the guinea pig inner ear. The detailed manufacturing process has been described by Farahmand Ghavi et al. (48). Briefly, the diameter of the rods ranged from 0.3 mm at the tip to 0.4 mm at the base, and insertion depth was 3 mm. Free PH EUR USP grade dexamethasone (C_22_H_29_FO_5_, Sanofi Chimie, Vertolaye, France) was homogeneously incorporated into the first 3 mm from the tip to the base (Figure 1). Based on *in vitro* release profiles (data not shown) three different drug concentrations were tested under *in vivo* conditions: 0.1, 1, and 10% drug load, which corresponded to a total amount of 0.55, 5.5, and 55 μg dexamethasone, respectively, in the implanted part of the silicone rod.

### Implantation of Silicone Rods

**Figure 2:**
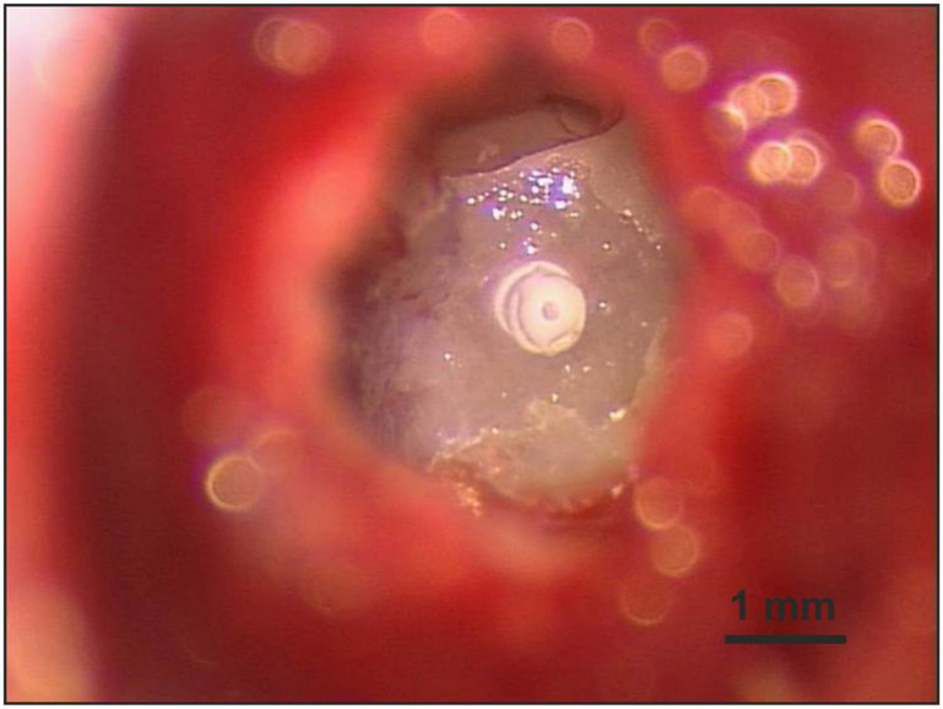
A cochleostomy (0.4 mm) was carefully drilled in the cochlear wall of the basal turn near the round window to obtain access to the scala tympani. The inserted silicone rod was fixed at the cochleostomy rim with histoacryl tissue glue.

To investigate the long-term pharmacokinetics under *in vivo* conditions, a total of 45 female albino guinea pigs (Dunkin Hartley, Charles River, Wilmington/USA) were used. Unilateral implantation of the silicone rod was performed under general anesthesia using a mixture of medetomidine (0.2 mg/kg), fentanyl (0.025 mg/kg), and midazolam (1 mg/kg) by intramuscular injection (thigh). Body temperature of the animals was maintained at 37°C on a heating pad. After local anesthesia (1% lidocaine), a 2-cm retroauricular incision was made with a scalpel parallel to the pinna. Muscle tissue was removed until the bone of the bulla tympanica was visible. The bulla was opened with a dental burr (1.4 mm) and then the hole was widened with a small bone rongeur. A small cochleostomy (0.4 mm) was drilled in the basal turn of the cochlea near the round window with a slowly rotating (150 rpm) dental burr (type 955908, Karl Storz, Tuttlingen/Germany). The electrode was slowly inserted into the scala tympani of the cochlea until the black marker on the rod was located at the rim of the cochleostomy. The insertion site was dried and closed with histoacryl tissue glue (B. Braun, Melsungen/Germany) around the silicone rod (Figure 2). The skin incision was closed with surgical sutures and histoacryl tissue glue. The scar was covered with liquid adhesive. After surgery, the animals received intramuscular injections of anesthesia antagonists into the thigh muscle: atipamezole 1.0 mg/kg + flumazenil 0.1 mg/kg + naloxone 0.03 ml/kg.

### Perilymph Sampling

**Figure 3:**
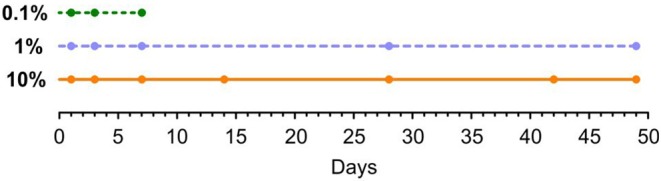
Time points of perilymph sampling for three different dexamethasone concentrations: 0.1, 1, and 10%. All animals were implanted at day 0.

Perilymph samples were taken at selected time points after implantation (Figure 3). For every sampling time point 3 animals were used. Perilymph sampling was performed under general anesthesia (see above). After additional local anesthesia (1% lidocaine) a ventral longitudinal incision was made on the head reaching from the nose to the caudal end of the jaw. After removing the jaw bone the remaining muscle on top of the bulla was deflected or removed. A hole was drilled into the bulla with a dental burr (1.4 mm) and widened with a small bone rongeur until the whole cochlea was visible. Perilymph samples were taken from the scala tympani using the method of apical sampling described in detail in Salt et al. (49). The cochlear apex was opened with a small fenestra pick (<0.1 mm) and the first 5 μl perilymph seeping from the small opening were sampled with a glass capillary (type 708707, Blaubrand, Wertheim/Germany) and diluted to 20 μl with double-distilled water and stored at −20°C. After sampling, animals were euthanized with an intracardiac injection of potassium chloride while still under deep anesthesia.

### Tissue Sampling and Analysis of Dexamethasone Concentration

After euthanizing the animal, the skull was quickly opened and the bulla was excised. The bone of the bulla around the cochlea was removed and the cochlea in combination with the petrous bone was briefly washed in isotonic phosphate buffered saline (PBS) to remove blood and tissue residuals and then stored under dry conditions at −20°C. The bone was mashed into powder and the dexamethasone was flushed out using methanol extraction. The extract was stored at −20°C. Dexamethasone concentrations were analyzed via HPLC-MS (Shimadzu with Sciencix detector API6500). The system was calibrated in a range of 0.02–10 ng/ml against D4-Dexamethasone standard.

### Analysis of the Dexamethasone Concentration in the Perilymph With Liquid Chromatography-Mass Spectrometry

To determine the concentration of dexamethasone, an LC-MS method was developed. Calibration was performed externally with pure dexamethasone (CAS 50-02-2). Chromatographic separation of 5 μl injected sample was achieved with an Agilent Zorbax Eclipse Plus C18 column (2.1 × 50 mm, 1.8 μm) and ZORBAX Eclipse Plus C18, 2.1 mm, 1.8 μm, UHPLC Guard Column at 20°C, with water with 0.1% formic acid (Mobile Phase A) and acetonitrile with 0.1% formic acid (Mobile Phase B). The elution gradient was as follows: 0–8 min, 2–40% B; 8–9 min, 40–98% B; 9–10 min, 98% B; 10–10.1 min, 2% B; 10.1–13 min, 2% B at a flow rate of 0.5 ml/min. Perilymph samples were analyzed in Multiple Reaction Monitoring (MRM) mode in positive mode using an Agilent 6460 LC-MS/MS system equipped with Agilent Jet Stream ESI Ion Source technology. The lower limit of quantification was 10 ng/ml. Collision energies for all respective dexamethasone fragments were optimized using an Agilent MRM Optimizer. Dexamethasone serial dilution solution and all perilymph samples were measured in random order. Data were reviewed and processed using Agilent Qualitative (v.B.07.00 SP1) and Quantitative Analysis (v.B.07.01 SP2), respectively.

## Results

**Figure 4:**
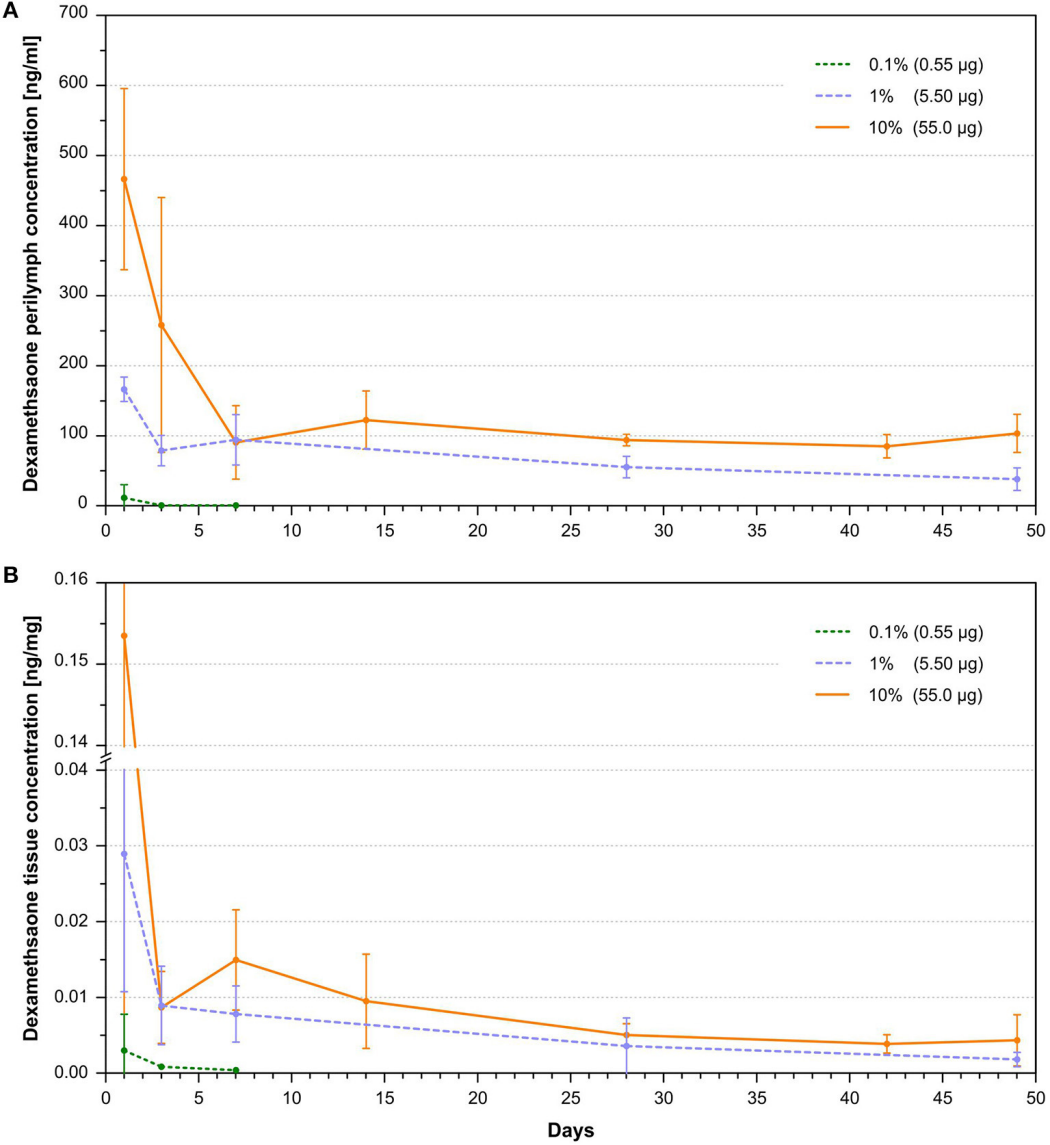
**(A)** Dependence of perilymph dexamethasone concentration in the scala tympani of implanted animals with 0.1, 1, and 10% loaded dexamethasone silicone rods on time after implantation (mean and SD, *n* = 3 per sample point). A burst release phase lasting 1–7 days, depending on silicone drug loading, was followed by a steady state phase characterized by constant drug concentrations. **(B)** Dependence of cochlear tissue dexamethasone concentration of implanted animals with 0.1, 1, and 10% loaded dexamethasone silicone rods on time after implantation (mean and SD, *n* = 3 per sample point). The concentration time course in tissue drug concentrations reflected the temporal dynamic seen in the perilymph dexamethasone concentrations. Measured concentrations in tissue were 2,000 times lower than perilymph drug concentrations.

**Figure 5:**
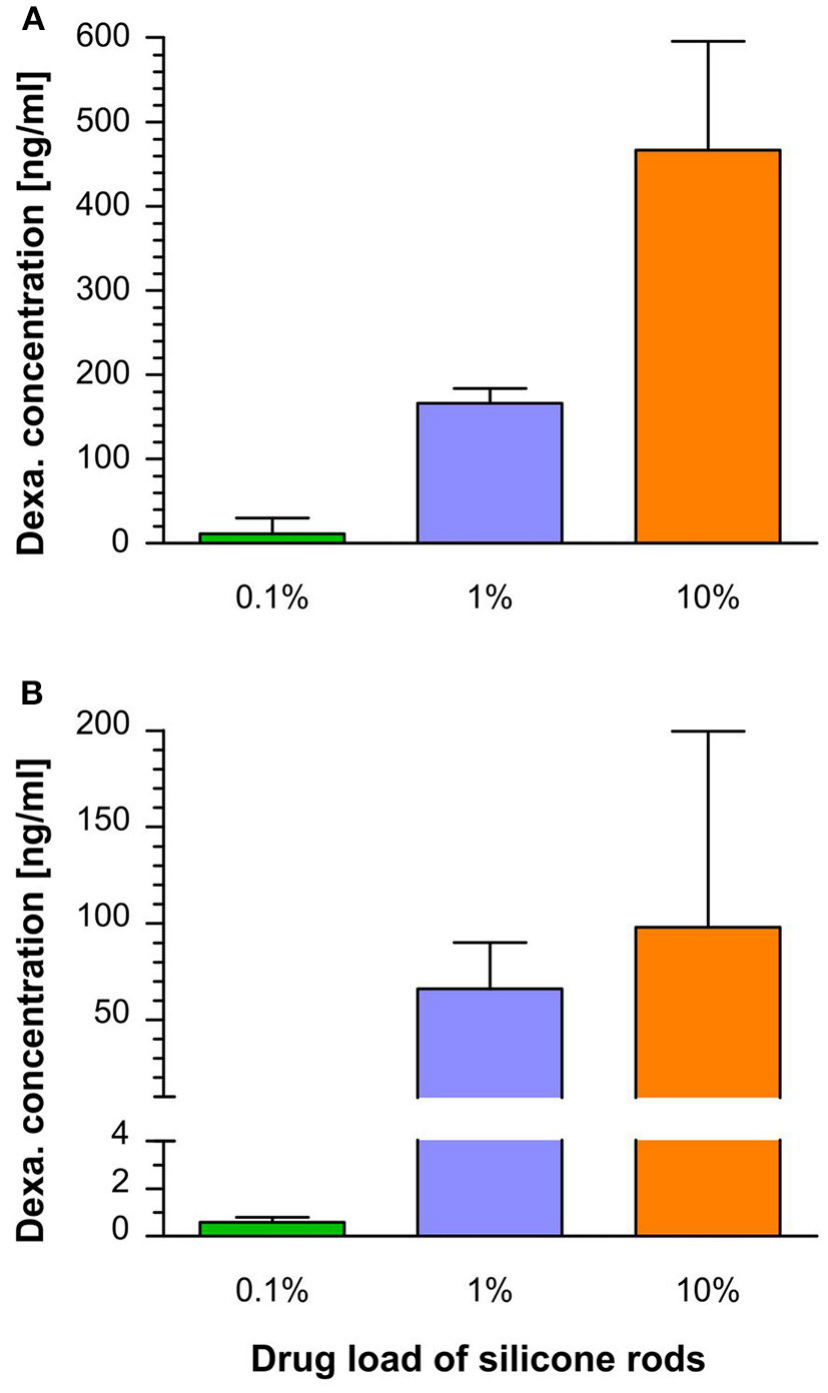
**(A)** Perilymph dexamethasone concentration in the scala tympani during the burst release phase (mean and SD) measured on day 1 after implantation for the three tested loaded rods (see Figure 4A). A higher drug load resulted in an increased burst release, showing an exponential tendency. **(B)** Perilymph dexamethasone concentration in the scala tympani during the steady state release phase (mean and SD). Calculation based on perilymph drug concentrations at the following time points: 0.1%: 3–7 days, 1%: 3–49 days, 10%: 7–49 days (see Figure 4A). The dexamethasone perilymph concentrations during the steady state release phase increased with higher drug load, showing a logarithmic tendency.

The dexamethasone levels in the perilymph for the 1 and 10% loaded rods showed an initial burst followed by stable concentrations during the observed time period (Figure 4A). A higher variance of dexamethasone concentrations was observed during the burst release than in the steady state phase. This is explained by a larger drug release variability of the silicone rods in the burst release phase as seen in their *in vitro* release kinetics (46). Higher drug loads resulted in an increased and longer lasting burst release showing a tendency of an exponential dependency from drug load (Figure 5A). The dexamethasone concentration for the 5.5 μg loaded rods was about 150 ng/ml during the burst release whereas for the 55 μg loaded rods a concentration >450 ng/ml was obtained (Figure 5A). Dexamethasone concentrations for the 1 and 10% loaded rods during the steady state phase were rather constant during 7 weeks of drug release after implantation. A drug load of 5.5 μg resulted in stable dexamethasone levels of about 60 ng/ml, whereas the loading of the silicone rods with 55 μg dexamethasone led to drug concentrations of about 100 ng/ml. The lowest dexamethasone loading tested (0.1%, 0.55 μg) resulted in low perilymph drug levels of about 0.5 ng/ml during the first days after implantation (Figures 4A, 5A). Therefore, the concentration time course resulting from this group was not followed until 7 weeks. A 10-fold increase of the dexamethasone concentration from 0.1 to 1% resulted in a 100-fold elevation of perilymph drug concentration during the steady state phase whereas the increase from 1 to 10% only raised the steady state drug level about 1.5-fold (Figure 5B). The dexamethasone perilymph concentrations during the steady state release phase seem to be dependent on drug load of the silicone rods with a logarithmic tendency. The dexamethasone concentration time course in cochlear tissue principally followed the same dynamic as was seen with respect to perilymph drug levels but with 2,000-fold lower concentrations (Figure 4B).

## Discussion

There are three main approaches to using the electrode carrier of a cochlear implant as a drug delivery device: (i) Integrating a fluid canal system into the carrier and combination with a catheter. The advantage is that the drug reservoir in the carrier can be refilled, but is accompanied by the risk of infection of the inner ear structures (20, 21, 50–52). (ii) Incorporating drugs into the silicone material or coating the carrier with drugs. This has the advantage of a closed system, but the drug amount is limited (42–44, 47, 53–55). (iii) Coating the electrode carrier with cells producing therapeutic molecules. The advantage of this approach is to be a source of complex macromolecules (56–60). However, stem cells are limited to the production of bioactive substances that are naturally produced in the body. This does not include synthetic or synthetically modified substances. Additionally, cells may release undesired substances and must survive the desired duration of drug treatment in the perilymph, an environment where no free cells are present naturally. The current study investigated the *in vivo* pharmacokinetics of electrode carrier dummies with homogeneously incorporated dexamethasone into the silicone material. Measurement of the dexamethasone concentration in the perilymph at different time points after implantation revealed that the dexamethasone-releasing silicone rods led to controlled drug levels with sustained dexamethasone perilymph concentrations over 7 weeks of implantation (Figure 4A). Furthermore, it was demonstrated that the drug level can be controlled by varying the initial dexamethasone concentration in the silicone. To achieve a close monitoring of the drug concentration time course, a setting of more sampling time points but fewer animals per time point were chosen. Although the small number of animals per time point imposes a risk for data interpretation, considering the small standard deviations at the different sampling points, we assume that the measured concentrations provide a good estimation of the drug levels in the cochlea. Within the examined range of drug concentrations, dexamethasone levels during the burst release increased with higher drug load, showing an exponential tendency, whereas the drug level during the following steady state phase characterized by constant drug concentrations depended in a more logarithmic manner on the drug load (Figure 5). This demonstrates the suitability of the manufactured silicone rods loaded with dexamethasone for use as an intracochlear drug delivery device. Importantly, there was no accumulation of dexamethasone in cochlear tissue since the tissue drug concentration time course shows the same dynamic as the perilymph drug levels, ensuring a controlled termination of the drug therapy (Figure 4B).

**Figure 6:**
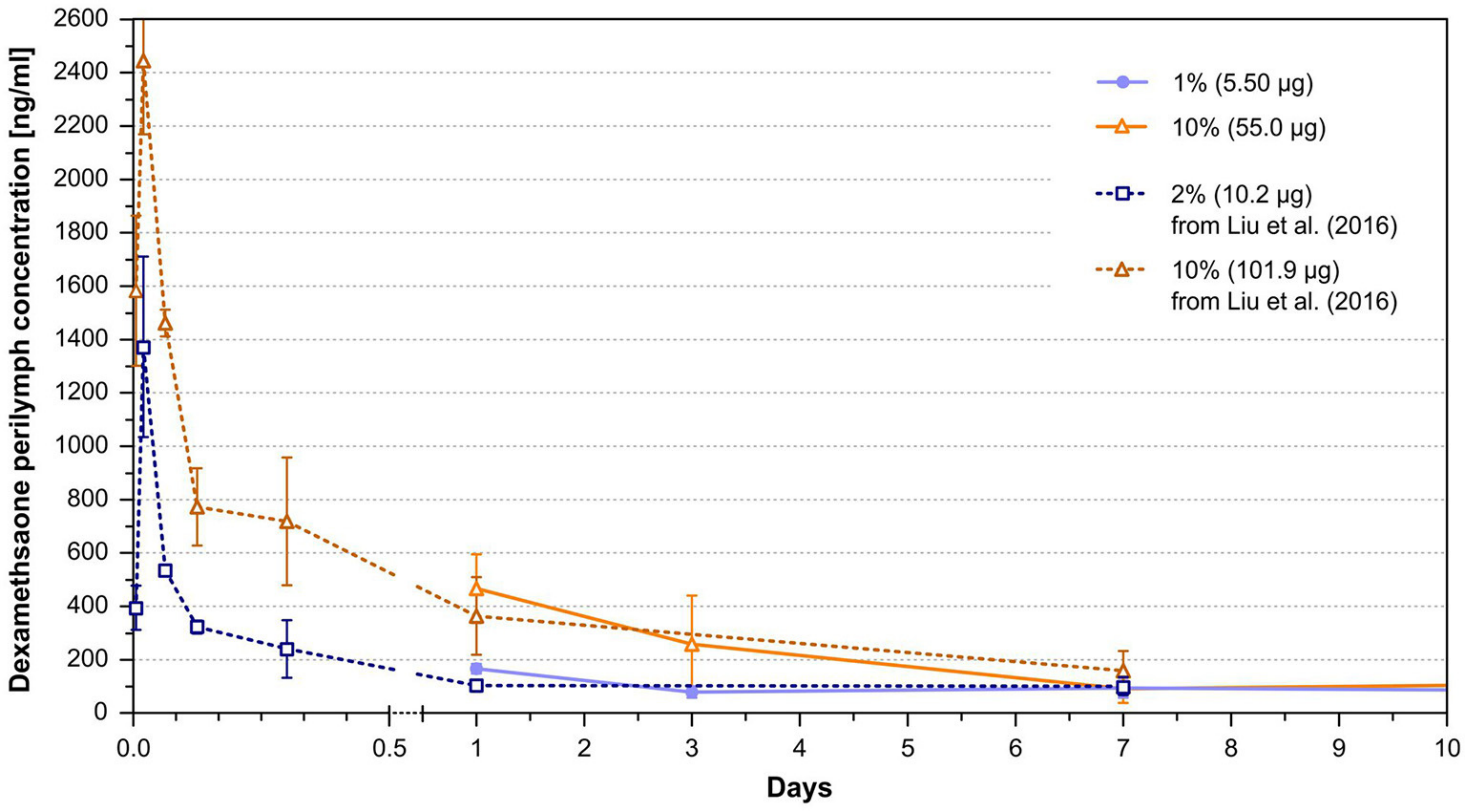
Perilymph dexamethasone concentrations of implanted animals with 5.5 μg (1%) and 55 μg (10%) loaded silicone rods after implantation from the current study taken from Figure 4A, and perilymph dexamethasone concentrations of animals implanted with 10.2 μg (2%) and 101.9 μg (10%) loaded silicone rods from the study of Liu et al. (46). The dexamethasone perilymph concentrations during the overlapping time interval of the studies are similar between the comparable rod loadings.

Several experimental studies have investigated the physiological effects after implantation of drug eluting electrode arrays. However, they have either not determined drug concentrations or have measured them only for a short time period. Many of those studies used similar drug eluting array dummies as the ones used in the current study. Thus, the pharmacokinetic data from the present study may be used to relate them to the measured biological effects. Liu et al. (46) investigated the pharmacokinetics of intracochlear drug delivery devices in a guinea pig model using fabricated silicone rods, similar to those used in the present study, containing 2 and 10% dexamethasone. The total amount of incorporated drug in the 3 mm inserted part of the rods was 20.4 μg for 2% concentration and 101.9 μg for the 10% concentration. In contrast to the rods used in the presented study, the rod diameter (0.5 mm) was consistent throughout the length and accounting for the higher total amount of dexamethasone. Liu et al. (46) measured similar dexamethasone concentrations during 1 week after implantation at overlapping time points of concentration measurement with the present study (Figure 6). In contrast to our study, the first sampling time point was 10 min after implantation. The high dexamethasone concentrations measured at 30 min after implantation (>1,200 ng/ml for the 2% and >2,400 ng/ml for the 10% rods) implied that the estimated peaks of the burst release in the present study are probably much higher than the data suggest since the burst release is already declining at our first sampling time point after 24 h. The authors have also noticed a steady state release phase following the burst release phase. However, our data show that the declining concentration in the time course of the 10% rod between 1 and 7 days, which was interpreted by the authors as slow concentration declining during the steady state phase, still partly belongs to the burst release phase, whereby the declining tendency can be explained (Figure 6).

The same group performed a study conducting physiological tests and histological examination after implanting the 2% dexamethasone loaded rods in guinea pigs in comparison to animals implanted with silicone rods without incorporated dexamethasone (55). Based on the data of the current study and total drug load of the rods used by Liu et al. (55), it could be speculated that the rod loading used in their study led to perilymph drug concentrations between 50 and 100 ng/ml during the steady state phase (Figures 4A, 6). Implanted animals from both groups displayed an initial ABR threshold shift after implantation. Animals from the dexamethasone group partly recovered from this functional hearing loss within the first 16 weeks of implantation. However, beyond this time point there was no further recovery, instead there was a slight tendency toward worsening the thresholds again in most frequencies. Thresholds of distortion product otoacoustic emissions (DPOAE) were protected from deterioration in animals implanted with dexamethasone eluting silicone rods until at least 16 weeks in comparison with control animals without dexamethasone treatment. Histological examination of the cochleae after 1 month of implantation revealed no difference with respect of formation of new fibrotic tissue within the scala tympani but expression of the inflammation factor TNF-α in cochlear tissue was lower in the dexamethasone group. A study by Farhadi et al. (61) using the same manufactured silicone rods containing 2% dexamethasone showed less infiltration of the cochlea with lymphocytes, macrophages, and giant cells after 3 days in animals implanted with dexamethasone eluting rods and a reduced lymphocyte, macrophage infiltration, and capillary formation at day 13 in comparison to animals implanted with only silicone rods.

Astolfi et al. (44) implanted silicone rods in guinea pigs containing 10% dexamethasone or without dexamethasone, with a total amount of incorporated drug of 86.7 μg in the first 3 mm from the tip. The rods had a conical geometry similar to the rods used in the current study, and the insertion depth was also 3 mm. Given that the total drug load was 86.7 μg and according to measured pharmacokinetics in 10% rods used in the current study containing 55.5 μg total dexamethasone, the rods probably led to drug concentrations >100 ng/ml during the steady state phase (Figure 4A). Within the first 2 weeks after implantation, CAP thresholds in animals from the dexamethasone group recovered whereas thresholds in animals from the group without dexamethasone treatment worsened further. The formation of new fibrotic tissue after 2 weeks was reduced in animals implanted with dexamethasone-eluting rods compared to animals implanted with no eluting silicone rods (44).

In a dose-response study by Bas et al. (45), several physiological and histological parameters were compared in a guinea pig model on insertion trauma between animals implanted with dexamethasone-eluting electrode carrier dummies and non-eluting dummies. In this study the same type of silicone rods with conically shape and drug loadings (i.e., total amount of dexamethasone) were used as in the current study, but perilymph drug concentrations were not measured. Although Bas et al. (45) used an insertion depth of 4 mm instead of 3 mm and silicone rods were equipped with an electrode, it could be assumed that the achieved drug levels were similar to the dexamethasone concentrations measured in the present study. Therefore, their physiological and histological data can be viewed in relation to the measured drug concentrations of the current study. They could show that a drug load of at least 1%, which corresponds to a dexamethasone perilymph concentration >50 ng/ml during the steady state phase (Figure 4A), is mandatory to protect hearing thresholds (ABR) in implanted guinea pigs. Although there was some protective effect on hearing thresholds for the 0.1% load corresponding to perilymph drug concentrations of 0.5 ng/ml (Figure 4A), the protection was incomplete. This result was also reflected by CAP threshold measurement. After 90 days of implantation for 1 and 10% loads of the dummies, thresholds were similarly low, whereas for the dexamethasone load of 0.1% thresholds were between the control group without dexamethasone and the other dexamethasone groups (1 and 10%) (45). Counting outer and inner hair cells revealed that the 0.1% drug load failed to protect hair cells in the basal and middle regions, whereas 1 and 10% loads protected hair cells almost completely. Histological examination of the preservation of synaptic contacts and nerve fibers was only performed for the 0.1 and 1% rod loads. Both dexamethasone loads had protective effects on synaptic contacts and nerve fibers, but for the 0.1% load this was incomplete (45). Therefore, the data suggest that a perilymph dexamethasone concentration of at least 50 ng/ml is necessary to protect neuronal structures in the cochlea from insertion trauma. Electrode impedance measurement over 90 days of implantation showed that all dexamethasone loads decreased impedance compared to animals implanted with dummies without dexamethasone although for the 0.1 and 1% drug loads there was a tendency for impedance to increase slightly starting at 1 week after implantation. Only the 10% loaded dummies showed no impedance increase until 90 days, which corresponds to a drug level during the steady state phase of about 100 ng/ml (Figure 4A). Histological examination for evaluation of fibrosis was only performed for 0.1 and 1% loads. For both loads, a reduction of newly generated fibrotic tissue was observed in comparison to animals implanted with dummies containing no dexamethasone. The effect appears to be more robust for the 1% load, although not statistically significant (45).

Wilk et al. (47) used the same silicone rod geometry and drug loads as the current study, only with the difference that the silicone rods had two electrodes. The rods were inserted 3 mm as in the current study. However, in order to increase insertion trauma, the silicone rods were inserted three times and removed twice before being left in the cochlea. They measured lower impedance after 90 days in animals implanted with both tested dexamethasone loads of 1% and 10% compared to animals with non-eluting silicone rods. However, there was no statistically significant difference between the 1 and 10% groups. The authors also observed a suppressive effect on the formation of new fibrotic tissue after 90 days in scala tympani of implanted animals with dexamethasone-eluting electrodes. This effect seemed to be more pronounced at the higher drug load (10%), although statistical significance was not reached. These results support implications based on the study of Bas et al. (45), that even low perilymph dexamethasone concentrations (0.5 ng/ml) are sufficient to have some suppressive effect on the formation of fibrotic tissue but the suppressive effect is more robust with higher drug concentrations in the inner ear fluid (50–100 ng/ml). The estimated dexamethasone concentrations relate to the mean concentrations in the fluid of scala tympani. It is known that local application to the cochlea will lead to non-uniform drug distribution within the inner ear, i.e., when substances are applied to the basal part of scala tympani, basal-to-apical concentration gradients in scala tympani perilymph have been demonstrated (62–64). Intracochlear drug delivery of small volumes results in a more uniform drug distribution then extracochlear (round window) application (65). However, even after longer intracochlear delivery time, basal-apical concentrations gradients remain (66). The present study measured the mean drug concentration in the fluid of scala tympani. Thus, these data cannot clarify which drug concentrations were actually reached in the apical regions. The presence and their degree of intracochlear drug concentration gradients within the cochlea with an intracochlear depot are not known and should be addressed in future studies with the final design of the drug releasing cochlear implant.

As pharmacokinetic data show during the burst release phase, there are much higher dexamethasone concentrations present in the perilymph of the scala tympani >1,000 ng/ml during the first hour after implantation and about 100 ng/ml during the first week measured for the 1% dexamethasone load (Figure 6) (46). It is unclear whether these high concentrations immediately after implantation are necessary for a protective effect, or whether a drug delivery device that achieves a drug level of 50 ng/ml from the beginning would have the same protective effect. Astolfi et al. (53) suggested that the main insertion trauma takes place during the first 2 days after implantation, when higher corticosteroid concentration may be necessary to protect the inner ear structures (53). On the other hand, it has been shown that dexamethasone concentrations >1,200 ng/ml begin to have toxic effects on outer and inner hair cells after 5 days incubation in an *ex vivo* cochlear explant model, possibly setting an upper therapeutic range for the use of the drug (67).

Data to evaluate the long-term effect of dexamethasone-eluting electrode carriers are still lacking. In the study of Bas et al. (45) for the implanted 1% loaded rods, the protective effect on hearing thresholds was unchanged through the end of the 13 week observation period. In the study of Liu et al. (55) using a 2% load, protection of hearing thresholds appeared to start to deteriorate at 16 weeks after implantation although ABR thresholds were still significantly different between treatment and control group after 24 weeks. Stathopoulos et al. (42) found no difference between two guinea pig groups implanted with either a control electrode dummy or a dexamethasone-eluting dummy in terms of spiral ganglia density, organ of Corti integrity, or fibrosis and ossification at 12 weeks after implantation. However, the lack of differences in hearing thresholds persisted during the whole observation period from the beginning. Douchement et al. (43) implanted drug-eluting electrodes containing 1 and 10% dexamethasone in gerbils and demonstrated preservation of hearing thresholds in both groups compared to animals with non-eluting electrodes at 6 weeks after implantation. However, after 1 year of implantation this effect persisted only for high frequencies.

On the other hand, clinical studies have shown positive effects on impedance and preservation of residual hearing for up to years even after single application of corticosteroids during cochlear implant surgery (28–31, 33). The dynamic of tissue reaction and degeneration of neural structures following cochlear implantation or other trauma may differ between rodents used in experimental hearing research and human beings. For example, in rodents a fast degeneration of spiral ganglia after loss of hair cells is seen within only a few weeks (17, 68–71). This seems not to be the case in human patients (12–15), in whom possible rehabilitation of hearing with the cochlear implant has been demonstrated even after years of deafness.

## Conclusion

Drug delivery devices made of medical-grade silicone for cochlear implants are suitable to achieve constant dexamethasone levels in the inner ear over several weeks. The drug concentrations in the inner ear fluid and cochlear tissue can be controlled by the dexamethasone load of the silicone rods. Results of this pharmacokinetic study, in combination with findings from studies measuring physiological parameters, suggest a therapeutic dose range between 50 and 100 ng/ml during the steady state phase for the CI insertion trauma model. A concentration of 50 ng/ml dexamethasone seems to be sufficient to protect the neuronal structures in the inner ear. For the prevention of fibrosis, already low dexamethasone concentrations show some effect; however, the suppressive effect increases with higher drug levels up to the highest tested concentration of 100 ng/ml. The impact of higher drug concentrations on insertion trauma during the burst release phase in the first days after implantation remains unclear.

## Data Availability Statement

All datasets generated for this study are included in the article/supplementary material.

## Ethics Statement

The animal study was reviewed and approved by Saxony-Anhalt state office for consumer protection and veterinary affairs, Germany.

## Author Contributions

AL: animal experiments, data analysis, and manuscript writing. SS: study plan and electrode dummies. KM, JT, and SB: study plan. IS: animal experiments. MK and BK: HPLC. SP: manuscript writing.

### Conflict of Interest

The study was financed by MED-EL Medical Electronics, Innsbruck, Austria. SS, KM, JT, and SB were employed by MED-EL at the time of the study. The study design was planned in consultation with MED-EL. MED-EL did not influence data analysis or publication of data. The remaining authors declare that the research was conducted in the absence of any commercial or financial relationships that could be construed as a potential conflict of interest.
